# The International Executive Committee of the Pan-American Medical Congress

**Published:** 1892-02

**Authors:** Charles A. L. Reed

**Affiliations:** Secretary-General; Cincinnati


### BULLETINS OF THE PAN-AMERICAN MEDICAL CONGRESS.

[OFFICIAL.]

### THE INTERNATIONAL EXECUTIVE COMMITTEE OF THE PAN-AMERICAN MEDICAL CONGRESS.

The Committee on Organization of the Pan-American Medical Congress, at its meeting at St. Louis last October, elected the following International Executive Committee: *The Argentine Republic*, Dr. Pedro Lagleyze, Beunos Aires; *Bolivia*, Dr. Emelio Di

Tomassi, La Paz; *Brazil*, Dr. Carlos Costa, Rio de Janeiro; *British North America*, Dr. Jas. F. W. Ross, Toronto; *British West Indies*, Dr. James A. DeWolf, Port of Spain; *Chili*, Dr. Moises Amaral, Santiago; *United States of Colombia*, Dr. P. M. Ibañez, Bogota; *Costa Rica*, Dr. Daniel Nuñez, San José; *Ecuador*, Dr. Ricardo Cucalon, Guayquil; *Guatamala*, Dr. José Monteris, Guatemala Nueva; *Haiti*, Dr. D. Lamothe, Port au Prince; *Spanish Honduras*, Dr. George Bernhardt, Tegucigalpa; *Mexico*, Dr. Tomas Noriega, City of Mexico; *Nicaragua*, Dr. J. I. Urtecho, Grenada; *Peru*, Dr. J. Casamira Ulloa, Lima; *Salvador*, Dr. David J. Guzman, San Salvador; *Spanish West Indies*, Dr. Juan Santos Fernandez, Habana; *United States*, Dr. A. Vander Veer, Albany, N. Y.; *Uruguay*, Dr. Jacinto De Leon, Montevideo; *Venezuela*, Dr. Elias Roderiguez, Caracas.

Hawaii, Paraguay, Santo Domingo, the Danish, Dutch and French West Indies are not yet organized. Nominations of local officers have been received from a majority of all the members of the International Executive Committee, and a number of the lists have been confirmed by the Committee of Organization. These will be announced as rapidly as acceptances are received.

CHARLES A. L. REED, *Secretary-General.*

CINCINNATI, January 15, 1892.

